# Digitization of neuropsychological diagnostics: a pilot study to compare three paper-based and digitized cognitive assessments

**DOI:** 10.1007/s40520-020-01668-z

**Published:** 2020-10-01

**Authors:** Antje Latendorf, Lina Marie Runde, Tiina Salminen, Anika Steinert

**Affiliations:** 1 Geriatrics Research Group, Charité-Universitätsmedizin Berlin, Corporate Member of Freie Universität Berlin, Humboldt-Universität Zu Berlin and Berlin Institute of Health, Reinickendorfer Straße 61, 13347 Berlin, Germany; 2 Synaptikon GmbH, Ritterstraße 3, 10969 Berlin, Germany

**Keywords:** Digitization of neuropsychological assessments, Paper- and tablet-based comparison, Cognitive impairment

## Abstract

**Background and objective:**

The number of people suffering from dementia is increasing worldwide and so is the need for reliable and economical diagnostic instruments. Therefore, the aim of this study was to compare the processing times of the neuropsychological tests Trail Making Tests A and B (TMT-A/B) and Color-Word Interference Test (CWIT), which were performed in both digital and paper versions.

**Methods:**

The pilot study was conducted among 50 healthy participants (age 65–83 years) using a randomized crossover design. The correlations and differences in the individual processing times of the two test versions were statistically analyzed. Further research questions concerned the influence of the individual usage of technology and the technology commitment of participants as well as the influence of the assessed usability on participants’ performance.

**Results:**

Between the two versions (paper-based vs. digital) statistically significant correlations were found in all tests, e.g., TMT-A *r*(48) = 0.63, *p* < 0.01; TMT-B *r*_s_(48) = 0.77, *p* < 0.001). The mean value comparison showed statistically significant differences, e.g., interference table (CWIT) *t*(49) = 11.24, *p* < 0.01). Correlations with medium effect were found between the differences in processing times and the individual usage of computer (e.g., *r*_s_(48) = − 0.31) and smartphone (*r*_*s*_(48) =  − 0.29) and between the processing times of the TMT-B and the usability (*r*_s_(48) = 0.29).

**Conclusions:**

The high correlations between the test procedures appear promising. However, the differences found in the processing times of the two test versions require validation and standardization of digitized test procedures before they can be used in practice.

## Introduction

The prevalence of neurodegenerative diseases, such as Parkinson’s or Huntington’s disease, has increased significantly. In this context, the most relevant clinical syndrome caused by neurodegeneration is dementia. An estimated 35 million people are affected worldwide [1]. While there is currently no way to prevent dementia, there are a variety of components available for diagnosing dementia. For reliable diagnoses, patients’ medical and medication history, physical status (e.g., hearing, vision, blood pressure, chronic conditions), laboratory tests (such as blood tests or brain scans), neurological tests (e.g., mobility, gait, sensory functioning), and neuropsychological tests (to assess cognitive functions) are required [2]. These assessments are typically carried out using paper-and-pencil-based methods. However, they are often complemented by using digital technologies supporting the calculation of scores, and administration or interpretation purposes. Therefore, the question arises whether a complete digitization of the mentioned assessments could be a useful approach. The benefits for a digitized approach are that it allows a more standardized execution without the testers’ influence and a high measure of reliability as well as an automated data collection [3]. Furthermore, Dahmen et al. [4] emphasize the benefits of digital technologies in relation to the growing demands on clinicians in times of demographic change and growing numbers of older people. Despite many advantages of technically supported assessments, a clinician who is educated and experienced in conducting neuropsychological assessments is irreplaceable [5]. Therefore, the early detection of cognitive impairment in the preclinical dementia phase is of utmost importance. The use of valid assessments and tools with high predictability, which are simple to perform and easy to implement in clinical routine, seems to be fundamental for the future.

A large number of computerized cognitive tests are available. In a systematic review, Wild et al. [6] described 11 computerized tests that assess or detect age-related changes in cognition.

A lot of studies have been conducted to investigate the differences between paper-based and digital neuropsychological tests. A pilot study by Snowdon et al. [7] compared the original, paper-based Montreal Cognitive Assessment (MoCA) with the electronic version (eMoCA) on 401 participants over the age of 18. This study found that 46.7% of participants who took the eMoCA had a final score less than 26, indicating mild cognitive impairments (MCI). In comparison, only 34.7% of participants who performed the MoCA paper-pencil-based had a score indicating an MCI [7].

Bonato et al. [8] used right-hemispheric stroke patients to test contralesional awareness and found computer-based testing methods to be more sensitive to subtle awareness deficits than paper-based alternatives. A standard neuropsychological test, the Trail Making Test was digitized (dTMT) and compared with the paper-based original on older adults (aged 50–93 years) by Dahmen et al. [4]. Besides measuring the processing time and the number of errors made, the dTMT also collects detailed timing information, pauses and lifts. Therefore, the dTMT captures more factors that might give rise to better results [4].

In a study by O’Halloran et al. [9] multiple cognitive tests were digitized given the fact that scoring paper-based tests is more error-prone than it is the case of automatically scored digital tests. In contrast to the study by Dahmen et al. [4], here the examiner was integrated into the computer-based testing, rather than being replaced by it. The study was conducted among 116 outpatients with schizophrenia. The results showed highly significant (*p* < 0.0005) measures of absolute agreement for all test comparisons [9].

The aim of this study was to identify possible relationships and differences in the processing times of the paper vs. digital versions and to examine the extent to which individual usage of technology and technology commitment, age and gender have an influence on this. In addition, we examined whether the individual assessment of the digital test usability was related to the difference in processing times.

The following research questions were central for the study:

1. Do the individual processing times of the paper-based and digital versions differ within the respective cognitive test procedure?
2. Do the factors individual usage of technology and technology commitment, and age and gender, influence the differences in processing time between paper-based and digital versions of the respective cognitive test procedure?
3. How do the test participants evaluate the digital version within the platform with regard to their usability? Is there a connection between an individual evaluation of the method and the difference in processing time between the versions of the cognitive test procedures?

## Materials and methods

### Participants

The sample included 50 persons aged 65–83 years (25 women, 25 men, *M*_age _= 72.7 years). The participants lived in the Greater Berlin area and were made aware of the study by flyers and notices at events. A total of 81 persons were interested to participate in the study. In the order in which they registered, 76 persons were screened by telephone. 26 of them did not meet the inclusion criteria and were, therefore, excluded, and the last five persons were not screened as the target sample size of *n* = 50 was already met. The participants involved fulfilled the following inclusion and exclusion criteria: they were at least 65 years old and had no legal support; they had presented neither severe affective nor cognitive disorders nor severe auditory, visual, linguistic, sensory, and motoric impairments in the last 2 years. In the same way, they did not show any serious systemic or cerebrovascular diseases or diseases of the central nervous system during this period. Participants were included in the study, despite the limitations mentioned above, if they had been symptom-free for more than a year due to an optimal drug therapy adjustment or if they had used suitable aids, such as glasses. The inclusion and exclusion criteria were chosen on the bases of the CERAD battery [10], which also includes the two Trail Making Tests.

As the participants did not receive any financial compensation for their expenses, they were offered their own cognitive test results from the study staff (psychologists) on site.

The participants were divided into two groups. The first group (paper-based version first, digital afterwards) consisted of 12 women and 13 men, the second group (digital version first, paper-based afterwards) consisted of 13 women and 12 men. The allocation to the two groups was made with a balanced gender ratio in mind, using the randomization function in MS Excel. Participants were pseudonymized. This means that each participant was assigned a unique participant number. Only the study staff had access to this assignment.

### Materials

The cognitive tests, Trail Making Test A and B (TMT-A and TMT-B) by Reitan [11] and the Color-word Interference Test (CWIT) by Bäumler [12], in paper-based form as well as in a digital version, were the subject of the investigation. While the paper-based version functioned as conventional test documents, the digital versions functioned as the test procedures on an online platform.

In order to examine further influencing factors, the participants answered a questionnaire on sociodemographic data and assessed the extent of their individual usage of technology (computer, tablet, smartphone, Internet) on a four-level rating scale from “frequently” to “never”. In addition, the participants answered the Short Scale of Technology Commitment of Neyer et al. [13]. This short scale consists of the three subscales technology acceptance, technology competence convictions and technology control convictions and is intended to predict the successful usage of new technologies, especially in old age. Each subscale consists of four items, each with five possible answers from "strongly agree" to "strongly disagree". "I am very curious about new technical developments." is an example of an item from the subscale technology acceptance.

The usability of the digital versions was assessed with a questionnaire consisting of five items from the System Usability Scale (SUS) by Brooke [14]. The original questionnaire consists of a 10-point questionnaire with five possible answers for the interviewees from “strongly agree” to “strongly disagree”. It enables the evaluation of hardware, software, mobile devices, websites, and applications. For reasons of test economy, only those items of the SUS were used whose answers were considered useful to the survey in terms of usability after this short period of application.

Furthermore, the four dimensions *stimulation, perspicuity, efficiency and dependability* from the User Experience Questionnaire (UEQ) by Laugwitz et al. [15] were used to assess the usability of the digital versions. The original UEQ contains 26 bipolar word pairs in a total of six dimensions, with which the user can describe their experiences in dealing with the digital application.

### Procedure

During the first telephone contact (Visit 0) the participants were informed about the study and checked regarding the inclusion and exclusion criteria. In terms of interest and suitability, a common date for testing was agreed upon and, at the same time, information for the test participant and a map with directions to the test center were sent by post. With the aim of optimizing both the organizational process and the instructions during the tests, a pretest was carried out with an additional participant (before Visit 1). For reasons of clarity, the test procedure is shown in Table 1.

**Table 1:** Sequence of assessments during the examination

	Group 1	Group 2
Visit 0	Telephone screening (inclusion/exclusion criteria, appointment)	
Visit 1	Participant information and consent, data protection guidelines	
	Cognitive tests	
	Paper-based:	Digital:
	Trail Making Test A	Trail Making Test A
	Trail Making Test B	Trail Making Test B
	Color-word Interference Test	Color-word Interference Test
	Questionnaire on sociodemographic data and individual usage of technology, short scale for recording individual technology commitment	
	Cognitive tests	
	Digital:	Paper-based:
	Trail Making Test A	Trail Making Test A
	Trail Making Test B	Trail Making Test B
	Color-word Interference Test	Color-word Interference Test
	Usability questionnaires	
	System Usability Scale	
	User Experience Questionnaire	

The testing took place during individual sessions. All participants received both paper-based and digitized versions of each cognitive test. The order of the tests (i.e., TMT-A, TMT-B, CWIT) remained constant, but the first group took the paper version before then taking the digital version, and this method was applied vice versa for the second group. Sequence effects were thus decreased. In order to reduce exercise effects, a paper-based questionnaire (including sociodemographic data, individual usage of technology, technology commitment) was used between the cognitive assessments. Subsequently, cognitive test procedures were repeated for the other, not yet completed, version (paper-based vs. digital). The participants were instructed according to the instructions of the test procedures.

**Fig. 1:**
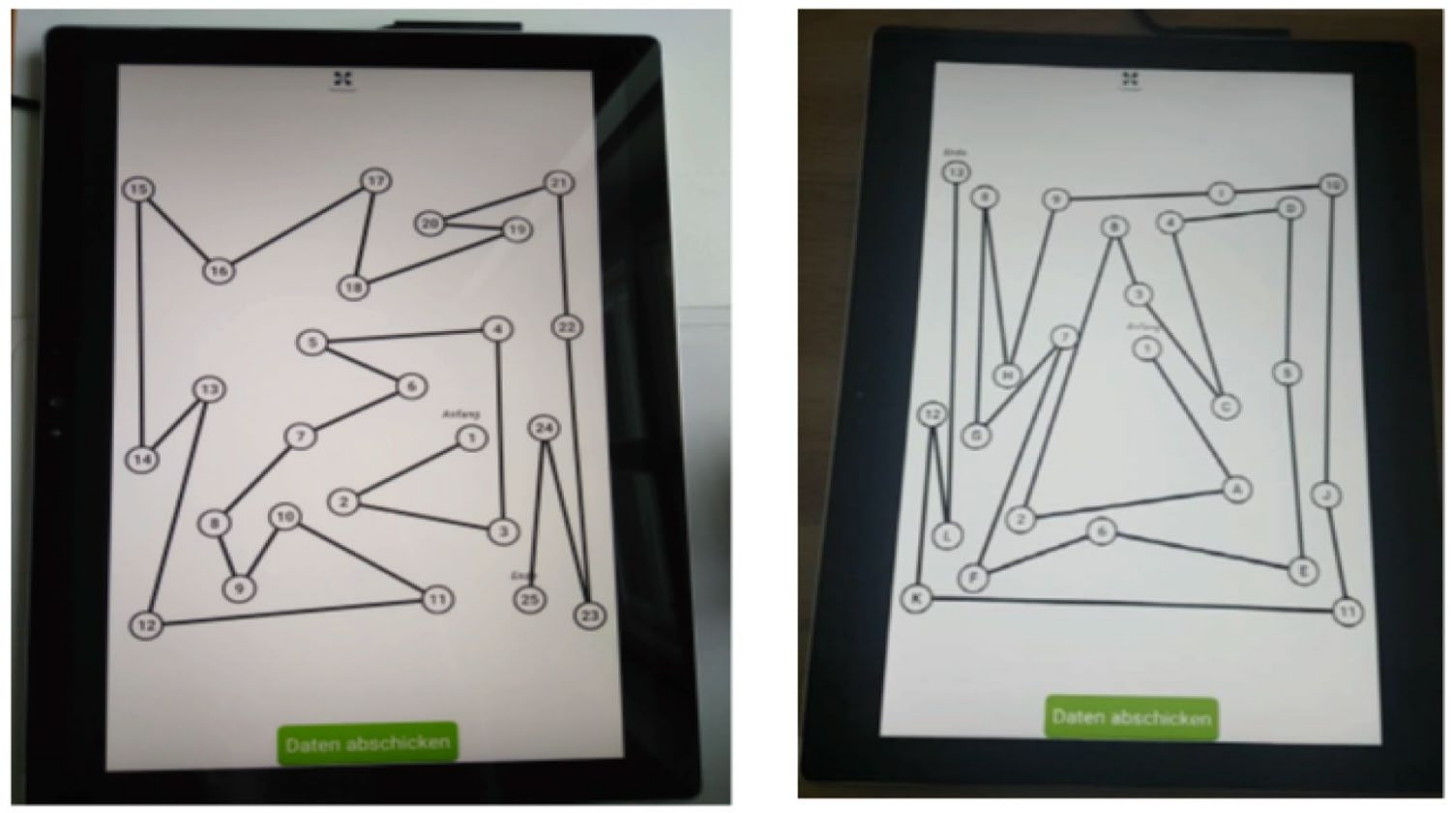
View of the digitally performed tests Trail Making Test A (left) and Trail Making Test B

The paper-based and digital version of the TMT differed relatively little. While the test participants used a pencil for the paper version, the test was presented on a tablet screen and a smartpen was used (see Fig. 1). The layout and the distances between the circles with the numbers (and letters) were almost identical in the digital and paper-based test version. Only the size of the presentation area (paper sheet vs. tablet screen) differed slightly.

**Fig. 2:**
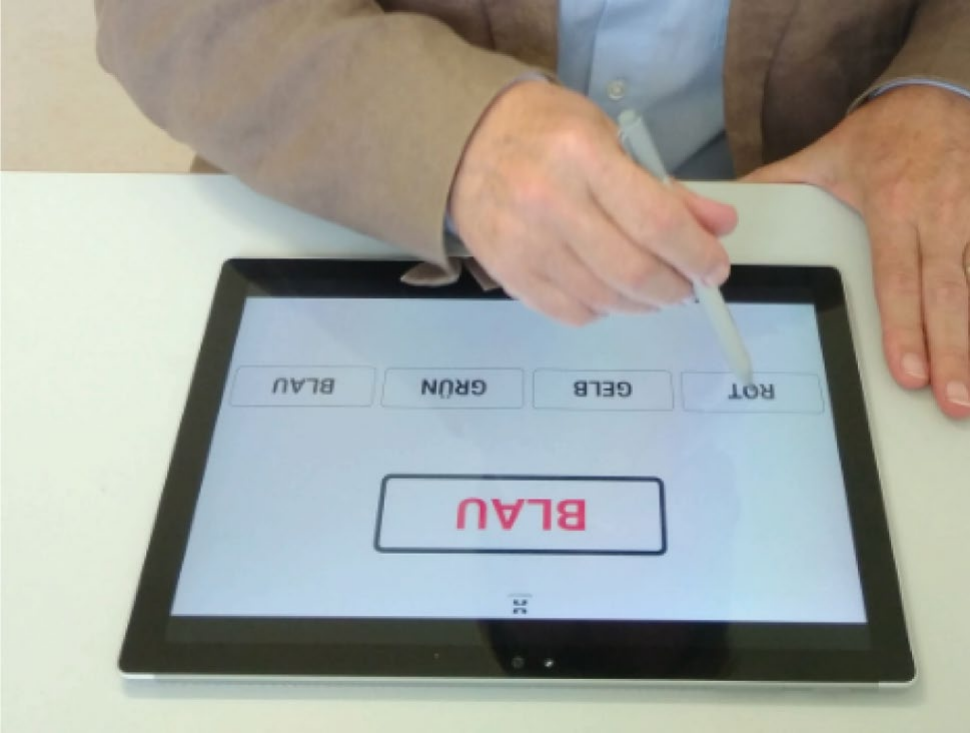
View of the digitally performed interference table of the Color-Word Interference Test

The types of CWIT implementation varied widely. In the paper version, conventional test panels were used. For the digital processing of the CWIT, the tables *color-word reading* (CWR) and *color-line naming* (CLN) from the official test by Bäumler [12] were presented as a PDF document on the tablet. The third *interference table* (INT) was available on an online platform (see Fig. 2).

Each participant first familiarized themselves with the task in a practice session. A color-word (e.g., “blue”) was presented with a color that did not match the content. The participant was then asked to choose the color that corresponded to the represented color of the color-word from four suggested colors. The words “blue”, “red”, “yellow” and “green” were displayed in small boxes under the color-word item which the participant was instructed to touch with the smartpen, as described above. A questionnaire to assess the usability of the cognitive test procedures to be performed digitally (consisting of five SUS items and four UEQ dimensions) completed the study. Here, too, the participants were instructed according to the instructions for the questionnaires used.

With a stopwatch the processing times for the cognitive test procedures in paper version were recorded and then noted on the protocol sheet. The digital versions were executed on a Microsoft Surface Pro 4 Tablet (12.2 inch) via the Chrome browser. In the study, only one tablet was used by all participants. Processing times were automatically measured by the system before being stored on a server and then transferred to Excel.

The data analysis was carried out both descriptively and inferentially with SPSS 24 statistical software. Mean value comparisons were carried out with a *t* test for dependent samples with the prerequisites of metric data level, normal distribution and variance homogeneity.

The Pearson product-moment correlation and the Spearman rank correlation were used to calculate the correlations. The use of the respective method was carried out according to the data level and taking into consideration the prerequisites (normal distribution and linearity of the data) and is indicated by the coefficients.

To avoid alpha inflation in the large number of tests, we carried out an adjustment according to Bonferroni-Holm (see Hemmerich [16]). In this case, the correlations were adjusted, since confirmation tests were performed here based on the assumed correlation. The mean value comparisons followed an exploratory question and were, therefore, not adjusted.

The correlation coefficients were interpreted as a measure of effect strength according to the conventions of Cohen [17] as follows: from 0.10 as small effect, from 0.30 as medium effect, and from 0.50 as large effect.

## Results

The sample comprised 50 participants, 56% of the participants were married or living in partnership, 30% were divorced or widowed and 14% were single. The participants lived either in a one-person household (50%) or in a two-person household.

When asked about the frequency of using technology, 90% of the participants stated that they “frequently” used a computer and 94% used the Internet “frequently”. All participants reported at least “occasional” usage of computers and the Internet and 60% of the test participants used a smartphone “frequently”. Nearly half of the participants (48%) had no experience with a tablet, while 36% reported “frequent” or “occasional” usage (see Table 2).

**Table 2:** Characteristics of the sample

Variables	
Age (M years, ± SD)	72.7 ± 4.07
Age (years)	in percent
65–70	32.0
71–75	38.0
76–80	24.0
81–85	6.0
Gender	
Male/female	50.0/50.0
Frequency of usage of technology (Internet/Computer/Smartphone/Tablet)	
Frequently	94/90/60/22
Occasionally	6/10/10/14
Rarely	0/0/8/16
Never	0/0/22/48
Technology commitment total score^b^ (M ± SD)	3.70 ± .58
Technology acceptance^a^	3.21 ± .83
Technical competence convictions	4.11 ± .61
Technical control convictions	3.78 ± .82

*N* = 50

^a^*N* = 49

^b^The scale ranges from 1 to 5

The sample achieved relatively high values on all scales in terms of technology commitment (total score *M* = 3.70, SD = 0.58). The scale ranged from 1 to 5 (see Table 2).

Research question 1: Do the individual processing times of the paper-based and digital versions differ within the respective cognitive test procedure?

### TMT-A: paper vs. digital comparison

**Fig. 3:**
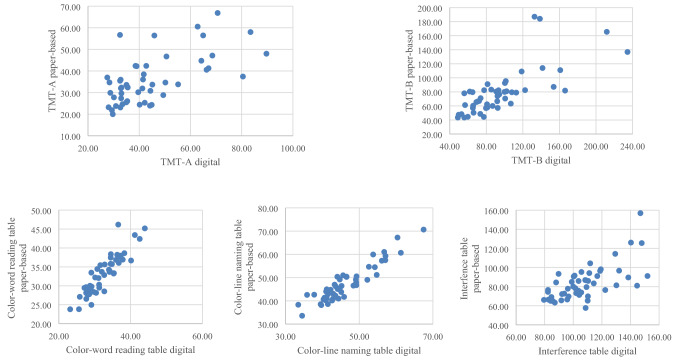
Correlations of processing times of paper-based and digital versions of the cognitive assessments (in seconds) (*N* = 50)

There was a statistically significant positive correlation between the individual processing times for the paper-based TMT-A, and the digital TMT-A (*r*(48) = 0.63, *p* < 0.01, adjusted). The longer the test participants needed to complete the paper-based TMT-A, the longer the digital execution took (see Fig. 3).

The processing time for TMT-A showed that the participants needed, on average, *M* = 8.53 s more for the digital version than for the paper version. In the mean value comparison, the processing times of the paper and digital version of the TMT-A differed to a statistically significant extent from each other (*t*(49) = − 4.90, *p* < 0.01). The key figures are shown in Table 3.

**Table 3:** Comparison of processing times of paper-based and digital versions of cognitive assessments (in seconds)

	Paper-based			Digitized			*t*(49)	95% CI		Correlation
Assessments	*M*	SD	Min–Max	*M*	SD	Min–Max		LL	UL	
Trail Making Test A	35.86	11.22	20.00–66.86	44.39	15.75	27.52–89.67	− 4.90**	− 12.01	− 5.03	0.63^a^
Trail Making Test B	78.66	32.42	43.45–187.09	94.41	39.70	39.13–234.43	− 3.36**	− 20.89	− 5.27	0.77^b^
Tables of Color-Word Interference Test										
Color-word reading	32.99	5.25	23.80–46.18	32.41	4.60	23.16–43.96	1.74	− 0.09	1.24	0.90^a^
Color-line naming	47.40	7.88	33.57–70.65	46.34	7.21	33.45–67.48	2.56*	0.23	1.90	0.93^a^
Interference	83.78	18.27	57.72–156.87	107.61	18.93	79.50–151.90	11.24**	− 28.10	− 19.58	0.68^a^

*N* = 50

CI = confidence interval; *LL* = lower limit; *UL* = upper limit. *t*-Test for dependent sample

*p < 0.05, two-tailed

**p < 0.01, two-tailed

^a^Pearson’s *r, p* < .01

^b^Spearman’s $\rho$, *p* < .001. *p* values of all correlations are adjusted

### TMT-B: paper vs. digital comparison

The individual processing times of the paper-based TMT-B and the digital TMT-B showed a statistically significant positive correlation (*r*_s_(48) = 0.77, *p* < 0.001, adjusted, no normal distribution). This means that the longer the paper-based processing took, the longer the digital processing took (see Fig. 3).

The processing time for TMT-B showed that the participants needed, on average, *M* = 15.75 s more to execute the digital version than the paper version. A comparison of the mean values showed a statistically significant difference (*t*(49) = − 3.36, *p* < 0.01) between the processing times of the paper-based TMT-B and the digital TMT-B. The requirement of normal distribution was not met, but could be ignored due to the sample size *n* > 30. The essential key figures can be found in Table 3.

### CWIT: paper vs. digital comparison

When determining the correlation coefficient of the processing times of the paper-based and digital version of the CWR tables, a significant positive correlation was found (*r*(48) = 0.90, *p* < 0.01, adjusted). A significant positive correlation (*r*(48) = 0.93, *p* < 0.01, adjusted) was also found for the processing times of the CLN tables. This also applied to the processing times of the INT tables (*r*(48) = 0.68, *p* < 0.01, adjusted). The longer the participants needed for the paper-based tables, the longer the processing of the digital tables took (see Fig. 3). In the descriptive evaluation of the CWR table, as well as for the CLN table, there was a slight time difference between the paper-based and the digital version. The INT table revealed a clear temporal difference. The participants needed an average of *M* = 23.83 s longer to perform the digital version (see Table 3).

The mean value comparison showed that the processing times of the CWR tables did not differ significantly from each other (*t*(49) = 1.74, *p* = 0.09), whereas the processing times of the CLN tables showed a statistically significant difference (*t*(49) = 2.56, *p* < 0.05). The mean value comparison of the processing times of the INT tables also revealed a statistically significant difference (*t*(49) = − 11.24, *p* < 0.01).

According to Cohen [17], all correlations of the different versions of each of the five tests can be classified as large effects.

Research question 2: Do the factors individual usage of technology and technology commitment, and age and gender, influence the differences in processing time between paper-based and digital versions of the respective cognitive test procedure?

### Correlation with usage of technology

The frequency of computer usage showed a negative correlation to the difference in the processing times (paper-based vs. digital) of the CWR tables (*r*_s_(48) = − 0.31). This means that the more frequently the participants used a computer, the smaller the difference was between the processing times of the paper-based and digitally presented CWR tables. The same effect could be seen in the smartphone usage and the difference in the processing times of the INT tables (*r*_s_(48) =  − 0.29). The frequency of smartphone usage was therefore, related to a smaller difference in the processing times of the paper-based and digital INT tables. According to Cohen [17], both correlations can be described as a medium effect.

**Fig. 4:**
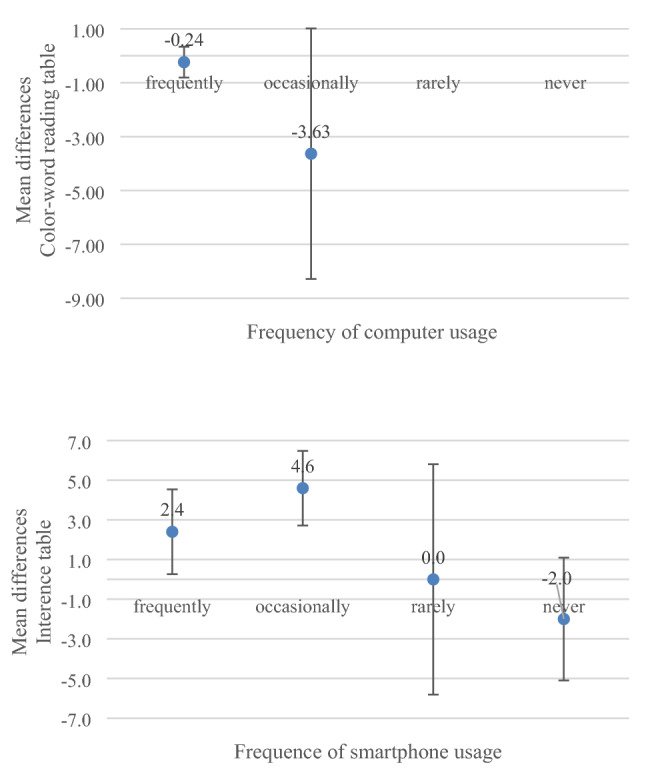
Mean values (and CI 95%) of differences in processing times of cognitive assessments and subjectively assessed usage of technology (in seconds) (*N* = 50)

The frequency of tablet usage showed no relevant correlation with the differences in processing times of the cognitive test procedures (see Table 4 and Fig. 4).

**Table 4:** Correlations between differences in processing times of cognitive assessments and subjectively assessed usage of technology and the dimensions of the User Experience Questionnaire

Variable	Individual usage of technology^a^			User experience questionnaire dimensions^b^			
	Computer	Tablet	Smartphone	Stimulation	Perspicuity	Efficiency	Dependability
Differences:paper vs. digitized versions							
Trail Making Test A	− 0.06	0.11	0.23	0.23	− 0.02	0.21	0.06
Trail Making Test B	− 0.10	0.05	− 0.04	0.38	0.18	0.40	0.30
Color-word reading table	− 0.31	0.10	0.25	− 0.10	− 0.14	− 0.30	− 0.19
Color-line naming table	0.27	0.03	0.18	− 0.09	− 0.06	0.03	− 0.03
Interference table	0.05	0.18	− 0.29	0.09	0.11	0.03	0.05

*N* = 50

*p* values of all correlations are adjusted

^a^Spearman’s $$\rho$$

^b^Pearson’s *r*

### Correlation with technology commitment

The subjective assessment of technology commitment by the participants on the scales technology acceptance, technology competence convictions and technology control convictions showed no relevant correlation with the differences in processing times between the two versions of implementation.

### Correlation with age and gender

The examination of the relationship between the differences in the processing times of the cognitive tests and the age and gender of the participants did not produce any relevant results. Neither age nor gender had any influence on the differences in processing times.

Research question 3: How do the test participants evaluate the digital version within the platform with regard to their usability? Is there a connection between an individual evaluation of the digital version and the difference in processing time between the versions of the cognitive test procedures?

### Correlation with subjective usability

**Fig. 5:**
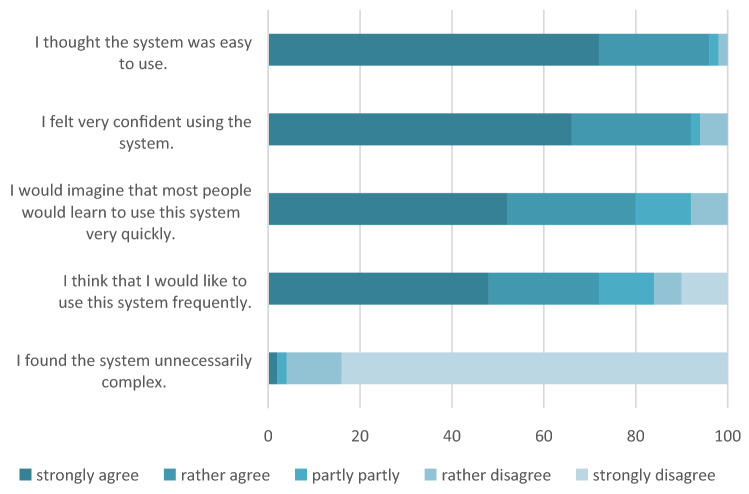
Evaluation of the usability/System Usability Scale items (in percent) (*N* = 50)

The participants’ assessment of the five items from the SUS shown in Fig. 5.

In response to the statement, “I thought the system was easy to use”, almost all participants (96%) agreed (“strongly agree” or “rather agree”) and 92% of the participants strongly or rather agreed with the statement regarding whether they felt confident using the system. The statement, “I think that I would like to use this system frequently”, was strongly or rather agreed by 72% of the participants and 96% of the participants strongly or rather agreed that the system was unnecessarily complex.

The examination of the relationship between the differences in the processing times for the cognitive tests (paper vs. digital) and the assessment of the usability of the digital version with the five items from the SUS showed a relevant effect. The statement, “I thought the system was easy to use”, correlated positive (*r*_s_(48) = 0.29) with the differences in the processing time for TMT-B (paper vs. digital). This means that the simpler the use of the system was felt to be by the participants, the higher the individual differences in the TMT-B processing times. According to Cohen [17], the strength of the effect is considered to be medium.

**Fig. 6:**
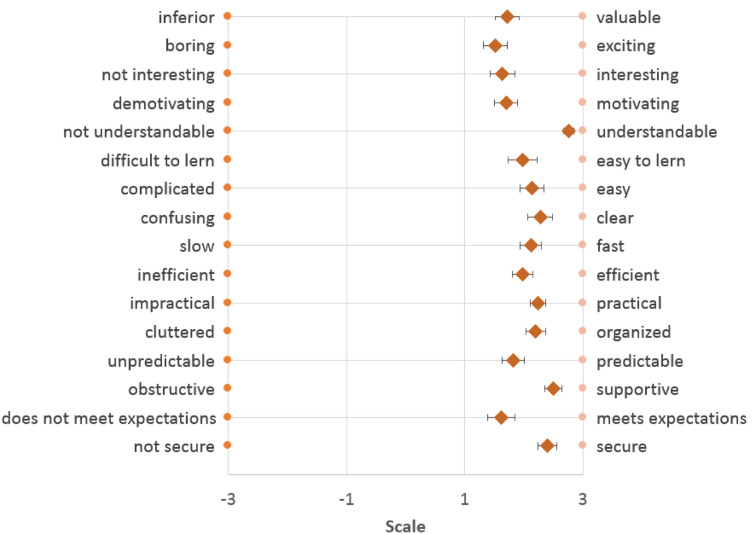
Evaluation of the usability/User Experience Questionnaire items (*N* = 50)

The results of the dimensions from the UEQ assessment (scale from -3 to 3) indicate a positive assessment of the usability of the digitized cognitive tests. The highest scoring was on the *perspicuity* dimension (*M* = 2.29). The dimension for *stimulation* was rated lowest (*M* = 1.68). The *efficiency* (*M* = 2.14) and *dependability* (*M* = 2.09) of the system were rated as approximately equal on average. The system was rated above average for the attributes *understandable*, *supportive* and *secure*. The attributes *exciting*, *meets expectations* and *interesting* received the lowest approval (see Fig. 6).

The examination of the correlation between the differences in the processing times of the cognitive tests and the assessment of the usability of the digital version with the four scales of the UEQ showed three relevant effects. The *stimulation, efficiency and dependability* dimensions correlated positively with the differences in the TMT-B (paper vs. digital) processing times (see Table 4). This means that the higher the usability of the digital version according to these three scales, the higher the individual differences in the TMT-B processing times. According to Cohen [17], these three correlations showed a medium effect.

## Discussion

The differences between paper-and-pencil-based and digital neuropsychological assessments were investigated in many ways. However, there is a gap in terms of influencing factors, especially when conducting digital assessments in dementia diagnosis. The aim of the presented study was, therefore, to identify factors that influence these differences. In previous studies, for example, it was found that age and gender influence the results (e.g. [18]). The presented study aims to examine whether the individual usage of technology (e.g., computer, tablet) has an impact on the individual processing time in the digital version due to the experience advantage and whether the processing time in the digital version also depends on the individual technology commitment. Ultimately, the usability assessment was a central aspect of the investigation, as it represents a key factor to ensure system acceptance in the target group of older people as well as a sustainable and ethically appropriate use in diagnostics.

The pilot study was conducted with 50 healthy participants who completed three cognitive test procedures (TMT-A, TMT-B and CWIT) both as a paper pencil test and as a digital test using a tablet.

In all test procedures, the individual processing times (paper vs. digital versions) correlated highly to a significant positive extent with each other. According to Cohen [17], the strength of all correlations between the respective test versions can be rated as large. This means that a high individual processing time of the paper version was accompanied by a high individual processing time of the digital version. It can be presumed that the correlation was based on the congruent cognitive requirements of the test versions. For example, the tests differed only in the input medium (pen and paper vs. tablet and smartpen) while the contents of the test procedures were transferred into the digitized equivalent and external factors were kept constant. This presumption has also been made by other authors, who attribute the correlations to constructs being recorded together (e.g., [19, 20]).

It also shows that the participants needed a statistically significant greater amount of time to complete the cognitive tests with the digital version than with the original paper version, with the exception of the CWR table. While completing the tests on paper presented the majority of the test participants with a new task (i.e., solving the respective test task), completing the tests on the tablet presented the majority of the test participants with two new tasks. On the one hand, a cognitively demanding task had to be processed, while on the other, the tablet had to be used as a more or less known medium, which resulted in an increased cognitive load in the information processing system [21]. This explains why the participants needed, on average, longer processing times for the tablet tasks (see also Drapeau et al. [22] [TMT-A]; [7] [MOCA]; [23] [CWIT]). The statistically insignificant difference in the processing times of the CWR table, which in comparison to the other tasks hardly differed in its digital version from the analog version, reinforces the argument of the double task. Snowdon et al. [7], who compared the paper-based execution of the MoCA with a digital execution (eMoCA), also found a significant higher processing time with the eMoCA, which they attributed to factors related to the use of digital devices or the user-friendliness of the software. In the present study, this could involve unfamiliarity in using the smartpen or possible reflections on the tablet screen, leading to a longer processing time.

The present study also revealed differences in the correlations of the individual test versions supporting this conclusion. TMT-A, which was performed first by all participants, correlated with *r* = 0.63 between the processing times of the two test versions. For the TMT-B, which was almost identical in execution to the TMT-A and was always performed immediately afterwards, this correlation between the test versions increased to *r*_s_ = 0.77.

Higher correlations in TMT-B than in TMT-A were also found by Fellows et al. [19]. A possible explanation could lie in the varying high demands between the two test procedures. While TMT-A primarily demands speed of perception, TMT-B additionally demands the more complex functions of inhibition and visual working memory [24]. In addition, TMT-B was performed by all participants always after TMT-A. Therefore, the similarity of the method and the resulting learning effect may have reduced the cognitive demands when performing TMT-B.

When considering the same tests as test repetitions (with different media) and comparing the effects found here with data from studies on test–retest reliability by other authors, the following becomes apparent: In a study with 484 participants over 50 years of age and a balanced gender ratio, the values *r* = 0.78 for TMT-A and *r* = 0.73 for TMT-B were found for the original paper-based tests [25]. The value of *r* = 0.63 found in this study is substantially lower, so that the difference could be attributed to the method. On the other hand, the value of *r*_*s*_ = 0.77 found in this study exceeds the value reported by Cangoz et al. [25] (*r* = 0.73). The value is just 0.04 higher, but due to the different media (paper-based vs. tablet) a lower value than in a test–retest study would have been expected. It is supposed that the already existing learning effect from the TMT-A could strongly compensate the cognitive effort by using the digital medium.

In the third test procedure (CWIT), a strong correlation was found between the test versions of the table CWR (*r* = 0.90) and CLN (*r* = 0.93). This is understandable insofar, as the paper version deviated only slightly from the digital version. In contrast, the two versions of the INT table differed more strongly, and a lower correlation of *r* = 0.68 was found. According to Cohen [17], these three effects are also to be rated as large. For the INT table, a visuomotor component in the form of the movement of the smartpen was also used for the digital conversion. It can, therefore, be concluded that manual response to an item, regardless of individual cognitive performance or technical experience, takes more time than is the case with the paper-based original version. Different processing times are to be expected when using a different response format. However, the direction of this influence seems to be undefined. Among others, Stodel et al. [26] found that less time was needed for verbal responses than for written ones. Overall, it can be concluded that the additional time required to operate the new technical medium (for most participants) was included in the processing time. As the participants belonged to a generation that had not grown up with computer technologies, they may have relied more on their experience with paper tests than with computer-aided tests (see Paul et al. [27]).

Bäumler [12] provides a global test–retest reliability of *r* = 0.91 for the overall profile of the three subtests of the CWIT. In contrast, the correlations found here are *r* = 0.90 (CWR table), *r* = 0.93 (CLN table) and *r* = 0.68 (INT table). The data for the first two tables, which hardly differed in their execution in both versions, are almost identical. However, the INT table differs. These data indicate a strong difference between the paper-based and the digital version and confirm the assumptions previously made when a learning effect is taken into account.

The examination of the extent to which the self-assessed usage of technology by the participants had an influence on the results revealed two relevant correlations. On the one hand, there was a correlation between the frequency of computer usage and a smaller difference in the processing times of the two versions of the CWR table (*r*_*s*_ = − 0.31). On the other hand, it was shown that the frequency of smartphone usage correlated with a smaller difference in the processing times of the INT table (*r* =  − 0.29). According to Cohen, the strengths of both correlations are medium or almost medium [17]. One, possible explanation for the advantage of frequent computer usage on the digital version of the CWR table is that it was the first test in the test order that did not require pen interaction and only required words to be read from a PDF document. In addition, the requirement of reading on a screen arises regularly for computer users, which may have resulted in a small advantage in the execution time of the digital CWR table due to this experience (see Mayes et al. [21]). With the CLN and INT tables, transformation tasks were added, which triggered a higher demand for executive functions. Here, the positive effect was no longer due to frequent computer usage, but to frequent usage of the smartphone. The INT table was the only (sub)test that required typing with a pen on the screen. Test participants, who already had experience with the operation of a touch screen and could, therefore, feel confident in using the interface, completed the digital version of the INT table somewhat faster. What was surprising was that the individual tablet usage had no effect on the processing time. The authors suppose that this influence was too small overall, since only one third of the participants stated that they used the tablet frequently or occasionally.

The technology commitment, with its subscales of technology acceptance, technology competence convictions and technology control convictions, and age and gender had no effect on the results. It is suggested that the sample was not sufficiently heterogeneous with respect to age in order to produce differences in performance. In addition, the participants were all retired and interested in technology, therefore, not enough variability in differences was expected.

One finding arising from the estimated usability of the digital method with the items from the SUS showed that the differences in the processing time for TMT-B increased the ease of use of the system as perceived by the participants (*r*_s_ = 0.29). The following reason can be offered for this unexpected result: The TMT-B had a much higher range than the TMT-A, i.e., the inter-individual differences between the processing times of the paper vs. digital versions were greater. However, the time required for processing may not be a reliable indicator of the difficulty of use. When dealing with digital systems/devices, time perception can be very subjective or unrealistic (e.g., Ball [28]). The fact that the test participants in this study found the system easy to use, despite the large differences (greater effort in completing the digital TMT-B which is shown in the duration of execution), can be explained as follows. It is possible that there may have been individual expectations that performing the (cognitive) tests, especially on the tablet, would be challenging and difficult.

This expectation was not confirmed in practice, which may have led to a feeling of "relief" that can be seen in the negative context of the more complex TMT-B. The fact that there are no other relevant correlations between the items in the SUS and the differences in processing times between the TMT-A and CWIT tests may be due to the fact that TMT-A is less complex and TMT-B was the 'middle' of the series of tests communicated to the participants.

### Limitations

All participants who took part in this study were already retired. In addition to the intended high average age, the inclusion and exclusion criteria meant that only persons with no significant physical or mental illness or cognitive impairment were included. Therefore, a selection bias can be presumed, and the sample should not be assessed as representative of the population. Furthermore, the sample size was small to medium, with the result that actual effects are not significantly represented due to the reduced statistical power. It is likely that the study was underpowered. Comparable studies [4, 27] have a similar sample size, meaning that, overall, the goal of obtaining initial results for the validation of digitized cognitive assessments seems to have been achieved.

The digital presentation of the CWIT, in particular task types of CWR and CLN, as presented to the participants in this study on the tablet as an image, should be modified in future experimental setup and be completely digitally processed. The INT table should also show fewer differences within the versions (verbal vs. manual response).

As all assessments were performed in the same session, the washout phase was considered too short by the study management. Exercise effects could,, therefore, not be excluded. In terms of economy and reasonableness for the participants, the study procedure was acceptable in its form, but must be discussed in a follow-up study. Likewise, we cannot exclude effects that have occurred due to the order of the test procedures.

The results of this study were compared with results from test–retest studies. However, apart from the different test versions used here, there usually lie several weeks between the test dates. In this study, however, all assessments were performed on one day of testing. These comparisons should, therefore, be viewed with some caution. Other studies similar to the present study should use a similar time interval for test–retest.

Of course, the use of other test methods would have been possible too. Especially qualitative methods may offer more information than quantitative methods. In this case we found the use of quantitative methods advantageous. SUS and UEQ are particularly suitable for the clientele investigated, as they are unproblematic to implement. They offer metric data level and the dimensions are easy to communicate.

Ultimately, the analysis of the test results was carried out with common statistical methods. Methods that focused more on the distribution of the data than on mean values would have been equally applicable.

## Implications

Due to the advancing digitization in all aspects of life, digitization in the health care sector is also being driven forward. This sector is particularly vulnerable due to its clientele and its particular characteristics. This study was conducted with healthy older volunteers who performed three cognitive tests in two versions. The results are multilayered and concern differences in implementation, different influencing factors and perceived usability. Further studies, in particular a validation study with patients, are already being planned.

The study presented here showed that paper-based and digital versions of all test procedures correlated highly with each other. The effects can be described as large. This is positive, although the processing times in the individual versions of the tests or tables differed widely. These differences, indicated by statistically significant differences in mean values, make it obvious that the digitized procedures can only be used with their own norm values for the diagnosis of cognitive impairment. The use of the existing paper-based norm values would lead to the cognitive performance of the patient being underestimated in most cases. This source of potential error must be avoided when introducing digitized test procedures.

A validation study with a sufficiently sized, representative sample of the population is indispensable. Any future study should play attention to heterogeneity with regard to age, educational attainment, state of health and previously diagnosed cognitive impairment. In order to generalize the results, focus should also be placed during recruitment on a non-university environment, the provision of expense allowances and the information transfer via analogue media. In spite of advancing digitization in all areas of society, the influence of individual technological experience and usage in the sense of validity and test fairness should be investigated on a larger scale in the development of digital cognitive assessments. It must be ensured that cognitive testing methods validly measure cognitive performance and that persons with little technological experience and/or commitment are not disadvantaged.
